# Cluster randomized controlled trial of Stepping Stones and Creating Futures to reduce mental health challenges among young men in informal settlements in KwaZulu-Natal Province, South Africa

**DOI:** 10.1177/00207640231174370

**Published:** 2023-06-05

**Authors:** Victoria Oyekunle, Andrew Tomita, Andrew Gibbs

**Affiliations:** 1 Department of Public Health Medicine, School of Nursing and Public Health, University of KwaZulu-Natal, Durban, South Africa; 2 Centre for Rural Health, School of Nursing and Public Health, University of KwaZulu-Natal, Durban, South Africa; 3 KwaZulu-Natal Research Innovation and Sequencing Platform (KRISP), College of Health Sciences, University of KwaZulu-Natal, Durban, South Africa; 4 Department of Psychology, University of Exeter, UK; 5 Gender and Health Research Unit, South African Medical Research Council, Pretoria, South Africa

**Keywords:** Young men, anxiety, posttraumatic stress, Stepping Stones and Creating Futures, informal settlement

## Abstract

**Background:**

Informal settlements are high density areas in and around cities, characterized by a lack of formal planning and basic amenities, being known in South Africa for high levels of mental disorder driven by violence, and complex social and economic challenges. In particular, young men’s poor mental health goes untreated, with relatively few evidenced-based interventions available in this setting.

**Aim:**

This cluster randomized controlled trial investigated the effectiveness of Stepping Stones and Creating Futures (SS/CF), a participatory gender transformative and economic empowerment intervention, on the mental health of young men living in South African informal settlement.

**Methods:**

A total of 674 young men ages 18 to 30 years were recruited in 34 clusters in Durban’s urban informal settlements. Clusters were randomly allocated (1:1) to either the experimental SS/CF or control arm and participants were followed-up over 24-months. Intention-to-treat analysis based on generalized estimating equations (GEE) were fitted to quantify the impact of SS/CF on the men’s anxiety and post-traumatic stress (PTS) symptomatology.

**Results:**

At end of the 24 months follow-period, anxiety (adjusted odds ratio [aOR]: 0.62, *p* = .04, 95% CI [0.39, 0.99]) and PTS (aOR = 0.52, *p* = .03, 95% CI [0.29, 0.93]) were significantly lower for group assigned to the SS/CF compared to the control group.

**Conclusion:**

SS/CF, a gender transformative and livelihoods strengthening intervention designed to address poverty and other socio-economic challenges in informal settlements reduced anxiety and PTS among men with mental health challenges living in informal settlements.

## Introduction

Poor mental health continues to be pervasive in sub-Saharan Africa (SSA; Agyepong et al., 2017), including in South Africa, where over 30% of South Africans have experienced a common mental health condition at some point in their lives (Stein et al., 2008). Conducted between 2003 and 2004, the South African Stress and Health (SASH) study, a national-representative study that consisted of a sample of 4,351 adults aged 18 years and above, showed that anxiety (a mental health condition for intense, excessive and persistent worry and fear that impair the ability to function in daily life (Perrotta, 2019) was the most prevalent lifetime mental health challenge (15.8%); (Herman et al., 2009). Post-traumatic stress (PTS), a debilitating mental health condition (e.g. nightmares or flashbacks, which emerge after experiencing or witnessing a terrifying event), had a national lifetime prevalence of 2.3% (Herman et al., 2009). The SASH study indicated the prevalence of anxiety and PTS to be 1.2% and 1.8% respectively in young people ages 18 to 34 years.

Informal settlements in South Africa are contextually distinct, initially being consequences of the country’s pre-democracy regime’s discriminatory migrant labour and spatial policy, they continue to persist. Previous studies indicate that these settlements face a range of health and social issues, including high crime rates and HIV (Corburn & Sverdlik, 2018; Gibbs, Reddy, et al., 2020), which are exacerbated by overlapping poor socio-economic conditions. South African studies have reported that young people living in informal settlements have increased levels of poor mental health, particularly anxiety (women = 18.6%, men = 19.6%; Mkhwanazi & Gibbs, 2021) and PTSD (women = 21.0%, men = 14.4%; Ndungu et al., 2020; Oyekunle et al., 2022).

The social, economic and physical settings in which people live greatly influence their mental health. Similar to other low- and middle-income countries (LMICs) (Alloh et al., 2018; Purgato et al., 2020), the contextual challenges faced by men in South Africa’s urban informal settlements are high, which puts them at risk for mental health issues. Contextual challenges include:

Economic distress: Poverty and a lack of job prospects are frequent experiences in informal settlements (Weimann & Oni, 2019). In these environments, young men may have trouble finding work, which could cause stress and anxiety over their finances. Poverty continues to have a crucial role in the relationship between economic disparities and a significantly elevated risk of mental illness (Anakwenze & Zuberi, 2013). While there are already high levels of mental health challenges in South Africa (Khasakhala et al., 2011; Williams et al., 2008), these can be further pronounced among young men living in poverty (Gibbs et al., 2016). In South Africa, and elsewhere in SSA, evidence points to a significant relationship between poverty and poor mental health (Burns, 2015; Skeen, Kleintjes, et al., 2010; Skeen, Lund, et al., 2010), including anxiety and PTS (Lund et al., 2010; Smith et al., 2005).

Crime and violence: Gang activity and other forms of violence are frequently found in high concentrations in urban informal communities (Drivdal, 2016; Gibbs et al., 2018). Mental health issues can become more likely when people are exposed to violence and criminality (Gibbs et al., 2016).

Social isolation: It might be challenging for young males to build social networks and contacts in informal settlements because of their density and lack of social infrastructure (Weimann & Oni, 2019).

Discrimination: There is a chance that young males in slum areas will be treated unfairly because of their social standing or other characteristics (UN-Habitat, 2015). Feelings of despair, low self-esteem and anxiety can be brought on by discrimination.

Limited access to healthcare: Being a common characteristic of informal settlements (Zerbo et al., 2020), young men may have difficulty accessing mental health services, making it challenging for them to seek treatment for mental health issues.

Trauma: Being exposed to traumatic experiences like natural disasters, neighborhood violence or conflict can have a long-lasting effect on mental health. Trauma has affected the lives of many young men who live in informal settlements (Gibbs, Hatcher et al., 2019). As a result of all of these factors, young men living in urban informal settlements may be more susceptible to developing mental health issues like anxiety and PTS.

Given the high levels of poor mental health among young men in urban informal settlements, likely driven by social circumstances, evaluating interventions that may promote positive mental health is critical (Rice et al., 2018). While McDowell et al. stressed the need to identify interventions that address the individual, interpersonal and social circumstances that drive poor mental health (McDowell et al., 2019), very few programmes have been developed and tested on young men living in informal settlements to establish their ability to improve mental health. There are two interventions in SSA worth noting. First, the SUUBI (meaning ‘hope’) programme, which was designed primarily to minimize HIV risk among AIDS-orphaned adolescents (Ssewamala et al., 2008), also assessed its impact on mental health (Han et al., 2013). The programme, which created savings account for children, provided workshops on financial management and mentorship and sought to minimize poor mental health among AIDS affected children, showed a significant reduction in hopelessness and depression levels (Han et al., 2013). Second, the Indashyikirwa intervention was implemented among couples to assess its impact on the reduction of intimate partner violence (IPV; Dunkle et al., 2020). The programme entailed engaging couples in a series of gender transformative curriculum that was layered onto a village savings and loans (VSLA) intervention and was effective in reducing IPV as well as symptoms of depression. While both interventions were beneficial, their application for young people living in informal settlements in South Africa remains unclear.

The Stepping Stones and Creating Futures intervention combined a gender transformative intervention (Stepping Stones) with a livelihoods intervention (Creating Futures), which sought to reduce perpetration of IPV in young men living in urban informal settlements (Gibbs et al., 2017). Gender transformative interventions aim to change deeply embedded gender norms, roles and expectations rather than merely reinforcing or maintaining them as a means of combating gender inequality and advancing gender equity. Livelihood interventions are economic empowerment tools used to deliver sustainable development by tackling the multifaceted problems of poverty. We conducted a secondary post-hoc analysis of this trial to investigate the intervention’s impact on mental health (i.e. anxiety and PTS).

Urban informal settlements are rapidly expanding globally and in South Africa, with over 850 million (York, 2015) and approximately 1.3 million people (Parliamentary Monitoring Group, 2019) living in them, respectively. Despite this large number, there is very little research on potential interventions that maybe applicable of young people, including the application for the Stepping Stones and Creating Futures (SS/CF) intervention to reduce anxiety or PTS disorder among men. In the main evaluation (Gibbs, Washington et al., 2020), it was found that after 24 months, men in the intervention group reported stronger livelihoods, reduced IPV perpetration as well as less alcohol consumption. The intervention may impact on men’s mental health by reducing poverty and alcohol use, and through the group spaces providing a sense of social connection. Hence, the present study examines the impact of SS/CF on young men’s mental health, specifically anxiety and PTS, in impoverished urban informal settlements of South Africa.

## Methods

### Study design

Using cohort data gathered at baseline and end-line for the main trial analysis of the impact of SS/CF among young men recruited into the study, we conducted a secondary post-hoc investigation. The original data set for the trial was provided by the research team allowing extraction of relevant variables.

### Study setting and population

This study entailed analyses of a cluster randomized controlled trial (RCT) that evaluates whether Stepping Stones and Creating Futures (SS/CF) reduces poor mental health among young men (18–30 years) in informal settlements. Clusters were identified by areas within informal settlements that were demarcated by pre-existing divisions or partitions such as natural barriers (e.g. a major road or waterway). The two-arm randomized controlled trial took place in urban informal settlements in Durban, KwaZulu-Natal Province and was conducted in collaboration with Project Empower (a local NGO that works to address gender-based violence). With over three million residents (eThekwini Municipality, 2018) and 500 informal communities (The Housing Development Agency, 2013), Durban is the largest city in the province that is at the heart of HIV and IPV epidemic in South Africa (Ramjee et al., 2016).

### Intervention and data collection

SS/CF is a community-based intervention originally designed to reduce IPV (although it has components that target mental health). The Stepping Stones component is focused on gender transformation and consists of 10 three-hour sessions (30 hours total), administered by trained peer facilitators to single-sex groups of 14 to 20 people, which was conducted over a period of 6 to 8 weeks. It employed participatory learning methods, such as critical thinking and incorporated the participants’ daily lives into the exercises. Among other topics covered, they targeted at improving social connections, this being the context in which actions occur while engaging with others. Creating Futures is focused on economic empowerment that consists of 11 three-hour sessions (33 hours in total) administered to the same single-sex groups as Stepping Stones. Setting livelihood priorities, dealing with crises, investing and saving, finding and maintaining jobs and balancing work aspirations were among the topics discussed, as reported elsewhere (Gibbs et al., 2022). There was a wait-list control, with no contact being made with the control participants other than to obtain data at the stipulated follow-up periods that is 12 months and 24 months. Data were collected independently on cellphones using the Mobenzi platform, which allowed for skip patterns and built-in logic checks, in either English, isiZulu or isiXhosa. Data were collected three times during the study, the first one being at baseline, the second at 12 months and the last at the end of the study after 24 months, from March to October 2018. A number of different retention strategies were created, including gathering information about participants’ friends and family in case we cannot reach them directly, sending text messages frequently throughout the follow-up, and enlisting the aid of the local community to better locate participants. The primary trial was funded by the ‘What Works to Prevent Violence Against Women and Girls? Global Programme’ which was a global programme funded by the UK Government’s Department for International Development (DFID). Detailed information on retention, adherence and trial funding can be found in the study protocol (Gibbs et al., 2017).

At the time of recruitment and informed consent, individuals were not blinded to the study arm. Despite the possibility of recruitment bias, participant welfare and project staff security were more at risk due to previous events of being promised support but not getting it and high levels of mistrust surrounding research, which are frequently found in marginalized communities.

### Randomization and sampling procedure

A cluster RCT, consisting of intervention and control groups (1:1 ratio), was conducted from September 2016 to October 2018. The intervention arm was administered with SS/CF, while the control arm was on a waitlist to receive the intervention after the final data collection. Once clusters were identified, randomization into intervention and control arms was carried out at the cluster level. Each cluster was assigned a cluster number, and the data analyst used a numbering system to randomize the clusters. When the numbers were randomly assigned to the clusters, the analyst was unaware of which cluster number belonged to which assignment group.

**Figure 1.**
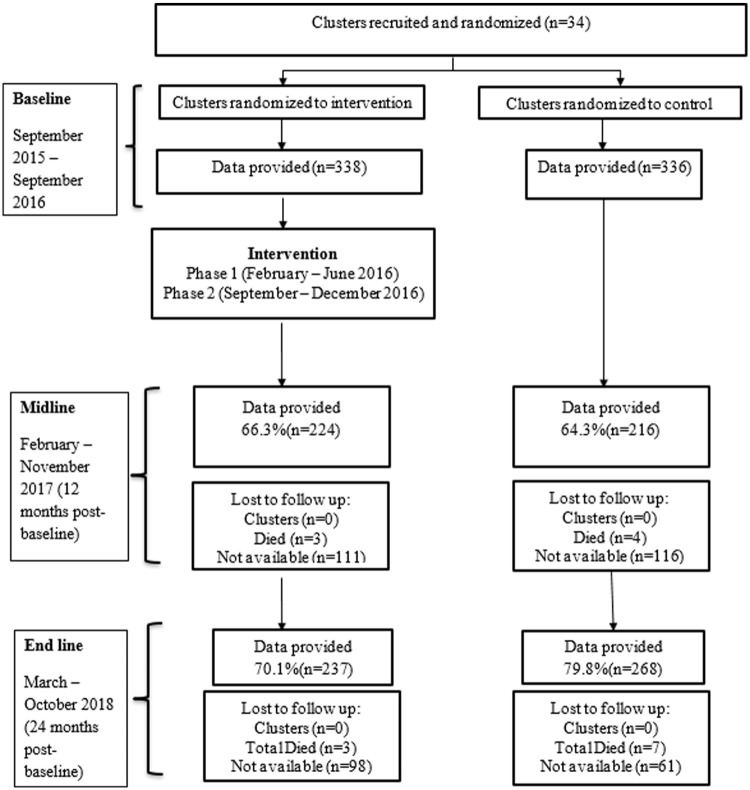
Stepping stones and creating futures CONSORT diagram for men. The flow diagram consists of a timeline that reports the enrolment, allocation, follow-up of clusters and study participants at each arm. 34 clusters were recruited and randomized to either the intervention (SS/CF) or control arm. After intervention was administered, follow-up was done on each arm after 12 months and after 24 months.

Project Empower workers identified potential study participants from clusters using snowball and purposive sampling techniques. The following inclusion criteria were used to enrol participants: (1) aged 18 to 30 years, (2) not currently in formal employment or education (3) resident within specified informal settlement clusters and (4) ability to communicate in English, isiZulu or isiXhosa. Those who (4) were unable or unwilling to provide written informed consent were excluded from participating in the study. Interested participants completed the informed consent form, with approximately 20 young men being sought from each of the 34 clusters (17 clusters in each of the arms). The informed consent consisted of the nature and purpose of the study, a detailed explanation of procedures to be followed and the risks, costs and potential benefits of involvement. Participants were made aware that their participation was voluntary, and that any information obtained from them would be kept confidential. Study participants were re-interviewed by professional research personnel who are proficient in English, isiZulu and isiXhosa at baseline, 12 and 24 months. The interviews were conducted using questionnaires that were forward and back translated from English into IsiZulu and IsiXhosa, with Figure 1 illustrating the flow chart of SS/CF. CONSORT guidelines for reporting cluster randomized trials were followed, with information on sample size calculation, randomization and study attrition being reported in the main outcome paper (Gibbs et al., 2019).

### Ethical consideration

The University of KwaZulu-Natal Biomedical Research Ethics Committee (BFC043/15) and the South African Medical Research Council Ethics Review Committee (EC006-2/2015) provided ethics approval to conduct the main trial. All participants provided written informed consent. The use of data for the present study was approved by the Biomedical Research Ethics Committee at the University of KwaZulu-Natal (00002795/2021). The trial was registered with ClinicalTrials.gov (NCT03022370).

### Measures

#### Outcome variables

Anxiety and post-traumatic stress were the main outcomes of the study, with two validated tools being used to measure the participants’ levels of both at 12 months and 24 months. Anxiety symptoms were identified by the 7-item General Anxiety Disorder-7 (GAD-7) scale that was developed to assess levels of anxiety over the past two weeks (Spitzer et al., 2006). Respondents were asked to choose from four possible responses in a Likert format, where 0 = not at all; 1 = for several days; 2 = over half the days; 3 = nearly every day, which were summed for each participant. Scores range from 0 to 21, this was recoded into binary, where; 0 to 4 = no anxiety symptoms and 5 to 21 = presence of anxiety symptoms. The internal consistency of the instrument, based on the Cronbach alpha, was 0.87.

The Harvard Trauma Questionnaire (HTQ), a 16-item measure validated in other South African populations (Machisa et al., 2017), was used to assess PTS symptomatology, with participants being asked about their post-trauma challenges during the previous week. The possible responses were based on a 4-point Likert scale, where 1 = not at all; 2 = a little; 3 = quite a bit; 4 = extremely.

The HTQ was recoded into binary variable where mean HTQ scores >2.5 were classified as having probable post-traumatic stress disorder [PTSD] (Mollica et al., 1992). The instrument’s internal consistency was 0.92 based on the Cronbach alpha. Data were obtained at baseline and follow-up after 12 and 24 months.

#### Baseline socio-demographic and other descriptors

In addition to socio-demographic information (e.g. age, marital status, education and employment status) and intervention assignment (i.e. study arm), six variables are worth noting for this current investigation, as they represent some of the challenges faced in informal settlements in South Africa: food security, crime participation, adverse childhood events, exposure to trauma event, gender equitable men scale and unhealthy alcohol use.

Food security was measured using the Household Hunger Scale (HHS), having been used in South Africa elsewhere (Hatcher et al., 2019). The three questions concerned food insecurity difficulties young men had faced in the previous month: 0 = never, 1 = rarely, 2 = sometimes and 3 = often, recoded into the three-level indication (none or little, moderate and high), with a Cronbach alpha for internal consistency of 0.83.

Crime participation entailed the use of the 10-item participation in crime measure, been validated and utilized in rural South Africa elsewhere (Gibbs et al., 2016), was revised to assess criminal engagement in the previous 12 months, with possible responses based on a 3-point Likert scale of 1 = never; 2 = once; 3 = more than once. The responses were grouped and recoded into two, response 1 being no = did not committed a crime in the past 12 months, while responses 2 to 3 were yes = committed a crime in the past 12 months. Internal consistency of the instrument based on the Cronbach alpha was 0.87.

Adverse Childhood Events (ACE) were evaluated using the 13-item scale Childhood Trauma Questionnaire (CTQ), having been adapted for use in South Africa (Machisa et al., 2016). Based on a 4-point Likert scale, respondents were asked about traumatic events encountered during their childhood, with possible responses: 1 = never; 2 = sometimes; 3 = often; 4 = very often. These were recoded into two categories, response 1 was denoted as no = never experienced it, while responses 2 to 4 were denoted as yes = experienced it. Scores ranged from 0 to 12 after recoding, and were further recoded into three, with 0 = no trauma, 1 to 3 as mild trauma and 4 to 12 as severe trauma. The instrument’s internal consistency was 0.86 based on the Cronbach alpha.

Exposure to trauma event was assessed through the revised Life Event Checklist from the PTSD Checklist, which has been validated in various South African communities (Machisa et al., 2017). The self-report instrument is an 8-item measure designed to assess for potentially traumatic situations in a respondent’s life. Participants gave yes or no answers to each question, totalling 8 responses, which were then recoded as 0 = no exposure and 1 to 8 = presence of exposure. Based on Cronbach’s alpha, the internal consistency is 0.72.

Gender Equitable Men Scale (GEMS) was used to measure masculinities and gender attitudes, being designed to generate evidence about the standard norms in a community as well as the benefit of any programme that can influence them. GEMS has been adapted in rural South Africa and used to measure gender attitudes (Wesson et al., 2022). Possible responses from participants in the 20-item scale included: 1 = strongly disagree, 2 = disagree, 3 = agree and 4 = strongly agree, with higher scores indicating less equitable attitudes. Internal consistency of the instrument based on the Cronbach alpha was 0.86.

Unhealthy alcohol use was screened by using the 10-item Alcohol Use Disorders Identification Test (AUDIT) scale (Saunders et al., 1993), which has been employed in informal settlements in South Africa [7]. (Ndungu, Washington, et al., 2020) The questioned relate to alcohol use in the previous year, with responses being based on a 5-point Likert scale: 1 = no, 2 = less than monthly, 3 = monthly, 4 = weekly and 5 = daily or almost daily. Scores were added and classified into two categories, where 0 = no harmful alcohol use and 1 = harmful alcohol use, the latter being classified with a score of 8 points or more. Based on the Cronbach alpha, the instrument’s internal consistency was 0.79.

#### Statistical analysis

First, we describe the baseline socio-demographic and clinical characteristics by study arm using descriptive statistical analysis. Second, we compared baseline socio-demographic and other baseline descriptors based on Chi-square to examine whether randomization was successful. Third, we conducted intention-to-treat (ITT) analysis on the men’s mental health outcomes at 12 and 24 months (mid-line and end-line) by fitting Generalized Estimating Equations (GEE) logistic regression models controlling for socio-demographic variables and baseline descriptors, setting anxiety and PTS as binary response with a logit link: g(µ_ij_) = log[µ_ij_/(1 − µ_ij_)], following the approach in the main trial analysis. We also undertook sensitivity analysis using multiple imputation as recommended given loss-to follow-up (Thabane et al., 2013) with 100 imputations hence increasing validity while preserving sample size and statistical power. STATA 17 was used for all analyses while accounting for the clustered nature of the data.

## Results

A total of 674 young men were recruited across 34 clusters between September 2015 and September 2016; with 338 (50.2%) being randomly assigned to the intervention group (SS/CF) and 336 (49.9%) in the control group clusters (Table 1). No cluster dropped out, although several participants did not complete the study, which resulted in the retention of 74.9% (*n* = 505) at 24 months, as shown in Figure 1. More details on recruitment and the full consort diagram are in the main outcome paper (Gibbs, Washington et al., 2020).

**Table 1.** Baseline socio-demographic and clinical characteristics by study arm.

	Overall	Intervention	Control
	% (*N* = 674)	% (*n* = 338)	% (*n* = 336)
Age group (years)			
18–19	10.7 (72)	8.6 (29)	12.8 (43)
20–24	51.8 (349)	53.9 (182)	49.7 (167)
25–29	30.9 (208)	32.8 (111)	28.9 (97)
30 and above	6.7 (45)	4.7 (16)	8.6 (29)
Employment status			
Not worked in the past 3 months	64.3 (433)	62.1 (210)	66.6 (223)
Worked in the past 3 months	35.7 (240)	37.9 (128)	33.4 (112)
Education status			
Primary/incomplete secondary	69.4 (468)	69.2 (234)	69.6 (234)
Secondary (complete)	30.6 (206)	30.8 (104)	30.4 (102)
Relationship status			
None	21.4 (144)	23.7 (80)	19.1 (64)
In a relationship	78.6 (530)	76.3 (258)	81.0 (272)
Food security			
Little/no hunger in the household	18.6 (125)	20.4 (69)	16.7 (56)
Moderate hunger in the household	56.5(380)	53.9 (182)	59.1 (198)
Severe hunger in the household	25.0 (168)	25.7 (87)	24.2 (81)
Crime status			
Never committed a crime	45.3 (305)	47.3 (160)	43.2 (145)
Committed a crime	54.8 (369)	52.7 (178)	56.9 (191)
Adverse childhood event (ACE)			
No trauma	7.7 (52)	7.4 (25)	8.0 (27)
Mild trauma	32.5 (219)	33.4 (113)	31.6 (106)
Severe trauma	59.8 (403)	59.2 (200)	60.4 (203)
Traumatic exposure			
No exposure	78.6 (526)	78.8 (264)	78.4 (262)
Presence of exposure	21.4 (143)	21.2 (71)	21.6 (72)
Gender attitude (GEM)			
Low equity	33.5 (225)	33.4 (113)	33.5 (112)
Moderate equity	36.6 (246)	35.8 (121)	37.4 (125)
High equity	29.9 (201)	30.8 (104)	29.0 (97)
Alcohol level (AUDIT)			
Sensible drinking	56.4 (380)	58.6 (198)	54.2 (182)
Alcohol problem	43.6 (294)	41.4 (140)	45.8 (154)
Post-traumatic stress			
No	85.6 (572)	85.9 (287)	85.3 (285)
Yes	14.4 (96)	14.1 (47)	14.7 (49)

*Note*. No baseline data available for anxiety.

### Baseline socio-demographic and clinical characteristics by study arm

At baseline almost two-thirds of the men were unemployed (*n* = 433, 64.3%), more than half only did primary education or did not complete secondary education (*n* = 468, 69.4%), while 56.5% (*n* = 380) and 25.0% (*n* = 168) experienced moderate and severe hunger, respectively (Table 1). For relationship status, 78.6% (*n* = 530) had a form of intimate relationship, nearly half (*n* = 294, 43.6%) reported harmful alcohol use. There was no difference between the two arms at baseline.

### Impact of SS/CF on mental health

Our analyses (Table 2) indicated that assignment to SS/CF was associated with a significant decrease in mental health symptoms after follow-up at 24 months compared to the control group for anxiety (adjusted odds ratio [aOR] = 0.62, 95% CI [0.39, 0.99]) and PTS (aOR = 0.52, 95% CI [0.29, 0.93]). At 12 months, while not significant, assignment to the SS/CF was associated with a decrease in mental health symptoms, compared to the control group in anxiety (aOR) = 0.79, 95% CI [0.36, 1.74]) and PTS (aOR = 0.56, 95% CI [0.24, 1.32]). Our sensitivity analysis confirmed the main findings (Table 3).

**Table 2.** Mental health outcomes at 12 and 24 months according to intervention based on intention-to-treat based on generalized estimating equation.

	SS/CF		Control		Adjusted odds ratio [95% CI]	*p*-value
	No of participants	Percentage	No of participants	Percentage		
PTS						
12 months	224	6.7	216	11.6	0.56 [0.24, 1.32]	.19
24 months	237	10.6	268	16.8	0.52 [0.29, 0.93]	.03
Anxiety						
12 months	224	43.3	216	44.4	0.79 [0.36, 1.74]	.56
24 months	237	50.6	268	61.6	0.62 [0.39, 0.99]	.04

**Table 3.** Mental health outcomes at 12 and 24 months according to intervention based on intention-to-treat based on generalized estimating equation after multiple imputation with 100 imputations.

	SS/CF		Control		Adjusted odds ratio [95% CI]	*p*-value
	No of participants	Percentage	No of participants	Percentage		
PTS						
12 months	338	33.7	336	35.7	0.56 [0.28, 1.12]	.10
24 months	338	29.9	336	20.2	0.58 (0.34, 0.99]	.05
Anxiety						
12 months	338	33.7	336	35.7	0.96 (0.64, 1.42)	.82
24 months	338	29.9	336	20.2	0.64 (0.44, 0.93)	.02

## Discussion

Our study examined the impact of SS/CF on young men’s mental health in urban informal settlements, the intervention being originally designed to reduce men’s perpetration of IPV (Gibbs et al., 2017). This study’s findings quantitatively demonstrate the beneficial effects of SS/CF on reduced anxiety and post-traumatic stress symptoms at 24 months.

There are three potential mechanisms through which SS/CF impacted on anxiety and PTS. The first possible explanation could be due to it being an economic intervention that significantly strengthened young men’s livelihoods thereby alleviating poverty. Research has shown a connection between unemployment and poor mental health (Thern et al., 2017); it may be that SS/CF’s impact on anxiety and PTS symptoms could be due to its effect on unemployment. Secondly, the qualitative data suggested that men found the groups beneficial, and reporting feeling less stressed and tense after the intervention (Gibbs et al., 2022). The importance of social support to reduce poor mental health, including anxiety and PTS has been shown in previous research (Price et al., 2018; Viseu et al., 2018). Thirdly, the intervention reduced alcohol use among the young men (Gibbs et al., 2022), with the reduction in anxiety and PTS possibly being mediated through this pathway. Despite change having begun at midline, changes were not significant then. The longer impact of SS/CF on poor mental health could be due to it requiring a longer period to have an influence on wage/earnings, as increased earnings positively impact mental health (Lund et al., 2011). Increased earnings and financial stability are likely to have occurred by 24 months, which may have played a role to mental health improvement.

Our investigation has several limitations, the first being that the evaluation of mental health outcomes was done using screening instruments, although it is unclear how screening measures would have impacted on the outcomes, as these were consistent across both arms. Secondly, this is a post-hoc data analysis of the intervention, which was primarily designed to improve IPV. Thirdly, the sampling techniques used (snowball and purposive) are forms of convenience sampling, which may negatively impact the generalizability of our findings. Data were also not collected for the number of individuals who were screened or refused to participate in the study. Individuals were not blinded to the study arm during recruitment, which might lead to recruitment bias. At the point of participant recruitment into the study, participants were aware which arm they were in, and this may have led to bias in recruitment. The lack of blinding was because the team were concerned that poor marginalized young people have often been promised support and not received it, and thus built-up resentment to broken promises. As such, there was no blinding. Despite attempts to locate participants, there was a difference in the follow-up by arm at end line, although multiple imputation was used to preserve statistical power and sample size. A higher attrition was detected among the intervention than the control group, although intention to treat analysis was used, where all participants were included in the final analysis. There are restrictions on how broadly our research can be generalized in other contexts, however this does not affect the validity of the results. The main outcomes paper describes the broader trial’s limitations (Gibbs, Washington et al., 2020). Notwithstanding these limitations, this is one of the few randomized studies that has demonstrated a significant reduction in poor mental health among young men in particularly challenging circumstances.

## Conclusion

This research on the community-based intervention for young men had positive findings on the potential of SS/CF to improve their mental health and social wellbeing, suggesting that working with groups of men is an important approach to reducing poor mental health in a context where people experience psychological disorders in disadvantaged settings. To establish the technique in the field of public health, this approach has to be adapted and replicated in various contexts outside of the initial research programme. It will also be useful to investigate the extent to which the influence of SS/CF persists over a longer period, and to explore similar strategies that could be administered in a shorter time. The effective reduction of mental disorder by Stepping Stones and Creating Futures will improve the quality of life of young men as they grow into adulthood. The intervention’s economic empowerment component will also give them skills to work with and equip them for their futures, which may continue to be challenging.
